# Adaptive multi-stage paramedian forehead flap for nasal reconstruction following HSV-related necrosis in a pediatric patient with primary immunodeficiency

**DOI:** 10.1080/23320885.2026.2641269

**Published:** 2026-03-11

**Authors:** Hatan Mortada, Shabeer Ahmad Wani, Doaa F. Andejani

**Affiliations:** a Division of Plastic Surgery, Department of Surgery, King Saud University Medical City, King Saud University, Riyadh, Saudi Arabia; b Department of Plastic Surgery & Burn Unit, King Saud Medical City, Riyadh, Saudi Arabia; c Division of Plastic and Reconstructive Surgery, Department of Surgical Specialties, King Fahad Medical City, Riyadh, Saudi Arabia

**Keywords:** Pediatric nasal reconstruction, forehead flap, paramedian forehead flap, HSV infection, immunodeficiency, alar necrosis

## Abstract

Large nasal defects in children are rare and create significant reconstructive challenges due to limited donor tissue, ongoing facial growth, and higher perioperative risks. These challenges are amplified in immunocompromised pediatric patients, particularly when infections such as herpes simplex virus (HSV) contribute to tissue destruction. The paramedian forehead flap is a reliable option for major nasal reconstruction in adults, but its use in children remains uncommon. We report a 4-year-old girl with an undiagnosed primary immunodeficiency and recurrent HSV infection who developed a full-thickness defect of the right nasal ala with partial auricular loss. A staged reconstruction was performed adaptively over five stages, including flap elevation with delay, transposition with native nasal skin lining and Matriderm application to reduce wound burden, partial division with alar sculpting, and final inset with eyebrow reconstruction. Intraoperative indocyanine green angiography confirmed adequate flap perfusion at each stage. The patient’s postoperative course was uneventful, and she demonstrated improved nasal airway patency, stable alar contour, and high parental satisfaction with cosmetic outcomes. This case suggests that a tailored, multi-stage paramedian forehead flap reconstruction may be safely performed in young children with complex nasal defects, including those with undiagnosed primary immunodeficiency and recurrent HSV infection, though further experience is needed to validate this approach. Careful staging, vascular assessment, and multidisciplinary collaboration were central to achieving successful functional and aesthetic results.

## Introduction

Pediatric nasal reconstruction represents a unique challenge in reconstructive surgery. Large nasal defects in children are uncommon and typically result from trauma, congenital anomalies, or infectious processes [1]. Unlike adults, children present additional considerations, including limited donor tissue, ongoing craniofacial growth, and higher risks of psychosocial distress related to facial disfigurement [2]. The rarity of extensive pediatric nasal defects means that literature on reconstructive strategies remains sparse, with most reports limited to isolated case studies or small series [3].

Among the available reconstructive options, the paramedian forehead flap has been established as the gold standard for major nasal reconstruction in adults [4,5]. Its reliability stems from the robust supratrochlear artery pedicle, generous skin paddle, and excellent color and texture match for nasal skin [5]. In children, however, its application is far less common due to smaller donor site dimensions, reduced forehead skin laxity, and concerns about potential effects on long-term facial growth [2]. Nevertheless, in cases of extensive full-thickness nasal tissue loss, the forehead flap remains one of the few options capable of restoring both form and function [6].

Infectious etiologies of pediatric nasal defects are particularly rare. While bacterial infections, such as Staphylococcus aureus or Pseudomonas aeruginosa, can cause destructive lesions, herpes simplex virus (HSV) has also been reported as a cause of necrotizing facial infections in immunocompromised children [7,8]. Recurrent HSV infections in the setting of immunodeficiency may lead to severe tissue loss, complicating both function and cosmesis. Management of such patients requires close collaboration between pediatric infectious disease specialists and reconstructive surgeons, particularly with respect to perioperative antiviral prophylaxis [9].

We present a rare case of a child with an immunodeficiency and recurrent HSV-related infections resulting in destruction of the right nasal ala. The patient underwent successful staged reconstruction using a paramedian forehead flap, highlighting both the feasibility of this approach in pediatric patients and the importance of multidisciplinary care in complex immunocompromised hosts.

## Case presentation

### Patient information

A 4-year-old girl, with no consanguinity, presented with progressive right nasal and auricular deformity. She was under active investigation for an undiagnosed primary immunodeficiency, with workup ongoing by the pediatric immunology service; a definitive diagnosis had not yet been established at the time of reconstruction. She also had failure to thrive. Neurological history included seizure disorder, right-sided weakness, and developmental regression beginning at 11 months, later showing partial improvement. MRI brain revealed a left parieto-occipital remote vascular insult.

### Preoperative evaluation

**Figure 1.**
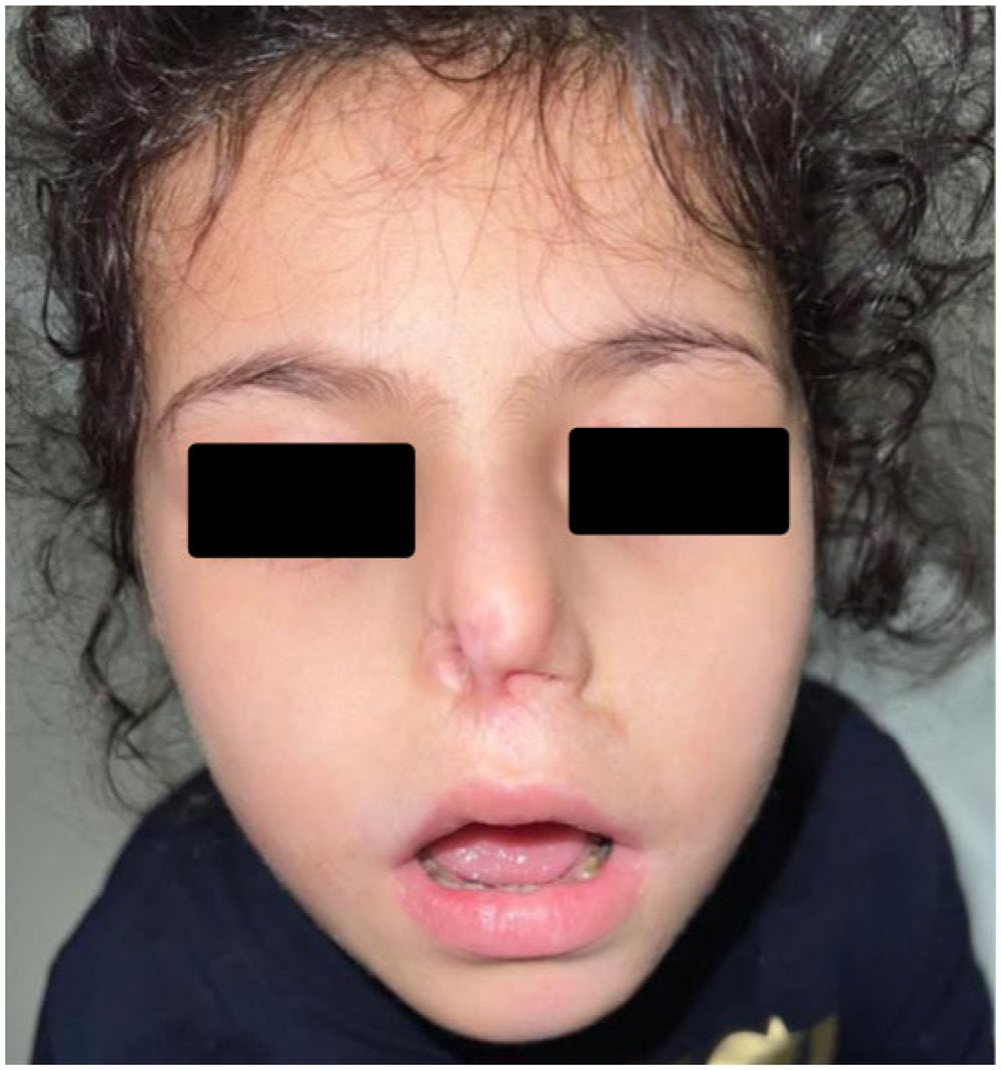
Preoperative view showing complete loss of the right nasal ala with columellar shortening and partial auricular deformity secondary to recurrent HSV infection in the setting of immunodeficiency.

Her history included recurrent severe infections, including pneumonia, otitis media, and confirmed HSV lesions. Recurrent herpetic facial lesions included one confirmed HSV-positive lesion that resulted in necrosis of the right nasal ala and auricle. She was maintained on suppressive antiviral therapy (Figure 1). She was treated with intravenous acyclovir, then transitioned to oral valacyclovir and maintained on suppressive therapy. On examination, there was complete loss of the right nasal ala involving skin, cartilage, and mucosa, shortening of the columella, and loss of approximately one-third of the auricle. Nasal obstruction was noted, causing difficulty in breathing. The patient’s undiagnosed primary immunodeficiency significantly influenced surgical planning. In coordination with pediatric infectious disease specialists, the following modifications were implemented: (1) an extended five-stage reconstructive protocol to minimize cumulative operative stress and allow close interval monitoring; (2) continuous suppressive antiviral therapy with valacyclovir maintained throughout all perioperative periods to prevent HSV reactivation; (3) minimizing foreign material introduction given the immunocompromised wound environment; (4) heightened surveillance for wound healing complications, flap compromise, and infection recurrence; and (5) multidisciplinary coordination with immunology, infectious disease, and neurology services at each stage.

### Surgical technique

A staged nasal reconstruction was performed by the plastic surgery team in coordination with pediatric infectious disease and neurology services. Rather than following a predetermined staging protocol, the reconstruction evolved adaptively, with additional stages incorporated as clinical circumstances required. Antiviral prophylaxis with valacyclovir was maintained throughout all perioperative periods.

**Figure 2.**
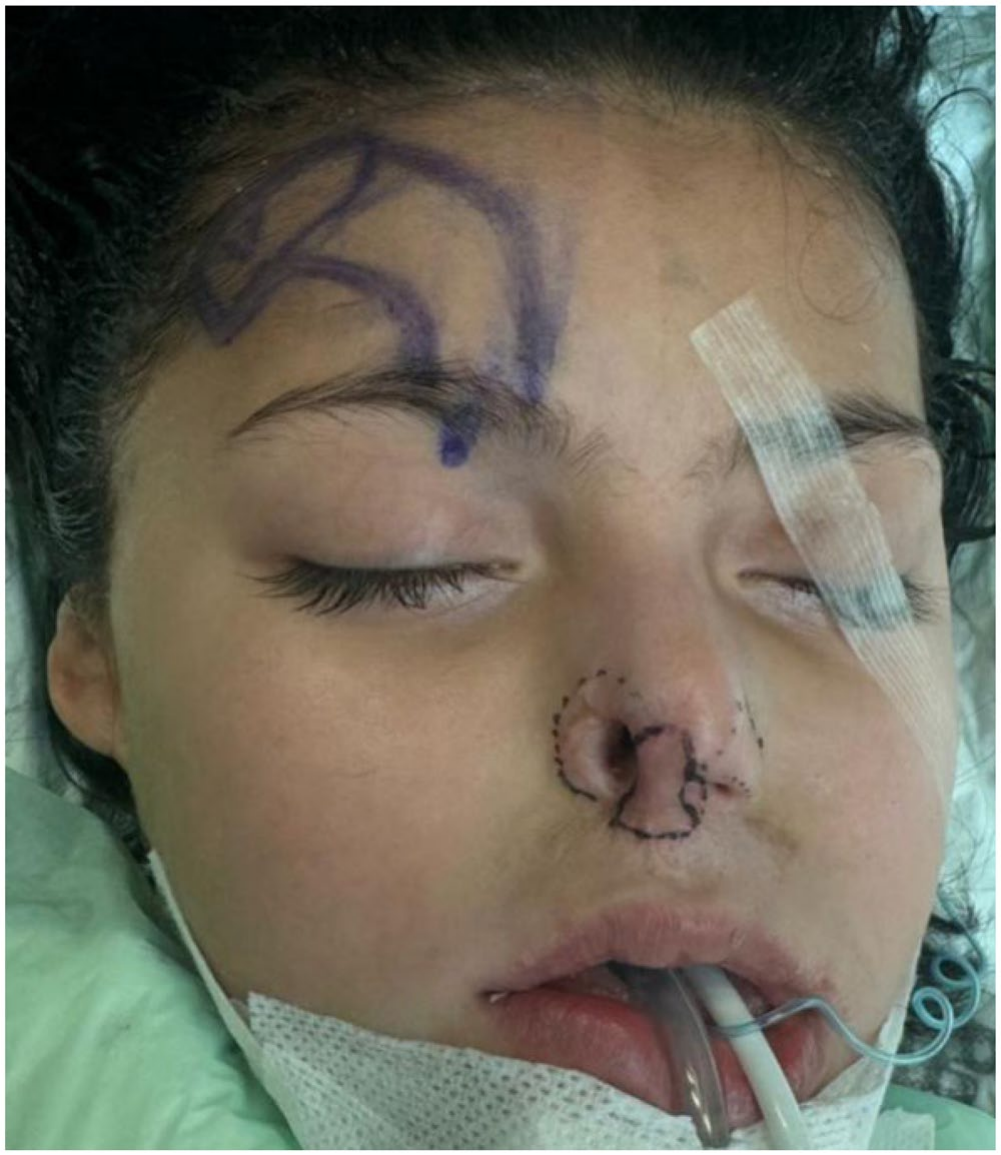
Intraoperative markings outlining the nasal defect and design of the planned paramedian forehead flap.

**Figure 3.**
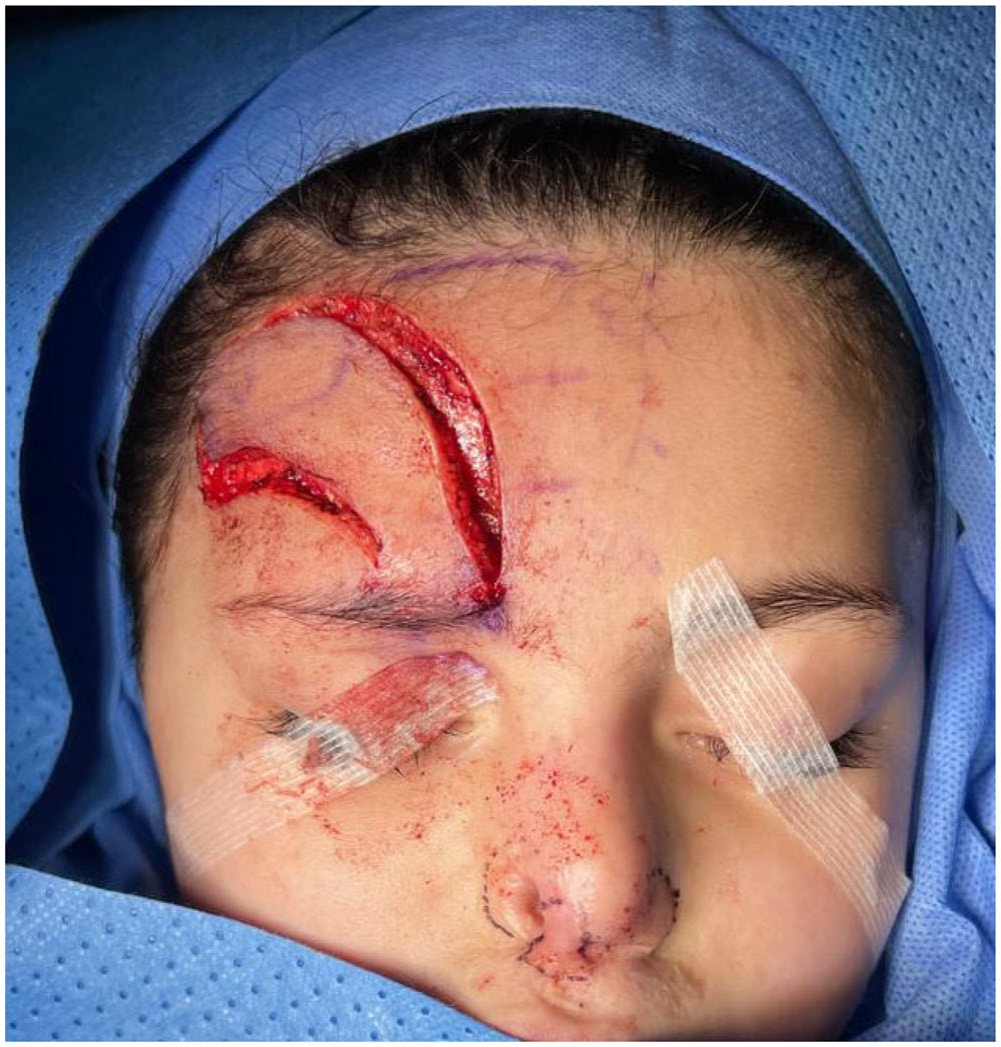
Elevation of the paramedian forehead flap based on the supratrochlear artery pedicle.

**Figure 4.**
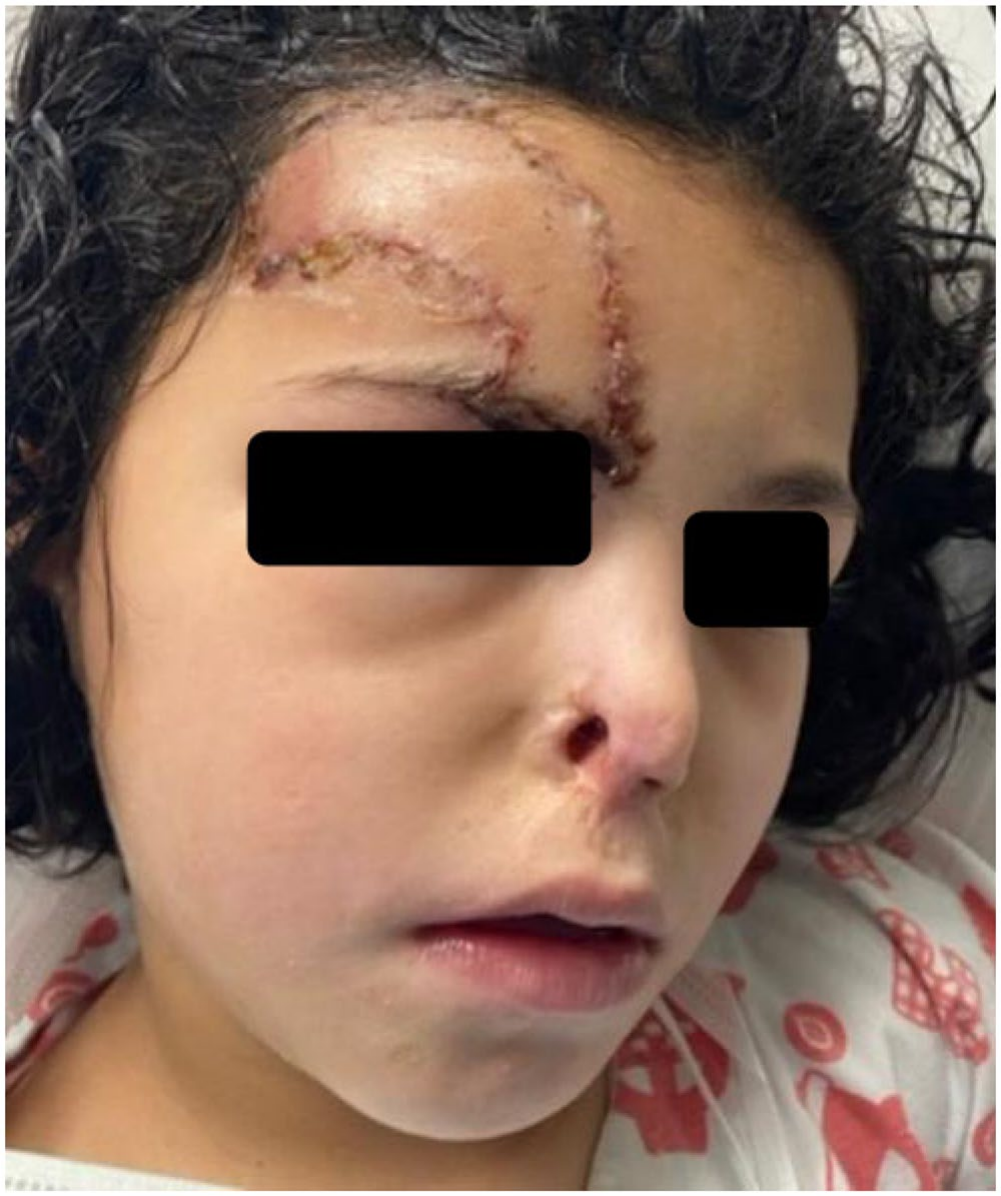
Early postoperative view of delayed paramedian forehead flap in place.

**Figure 5.**
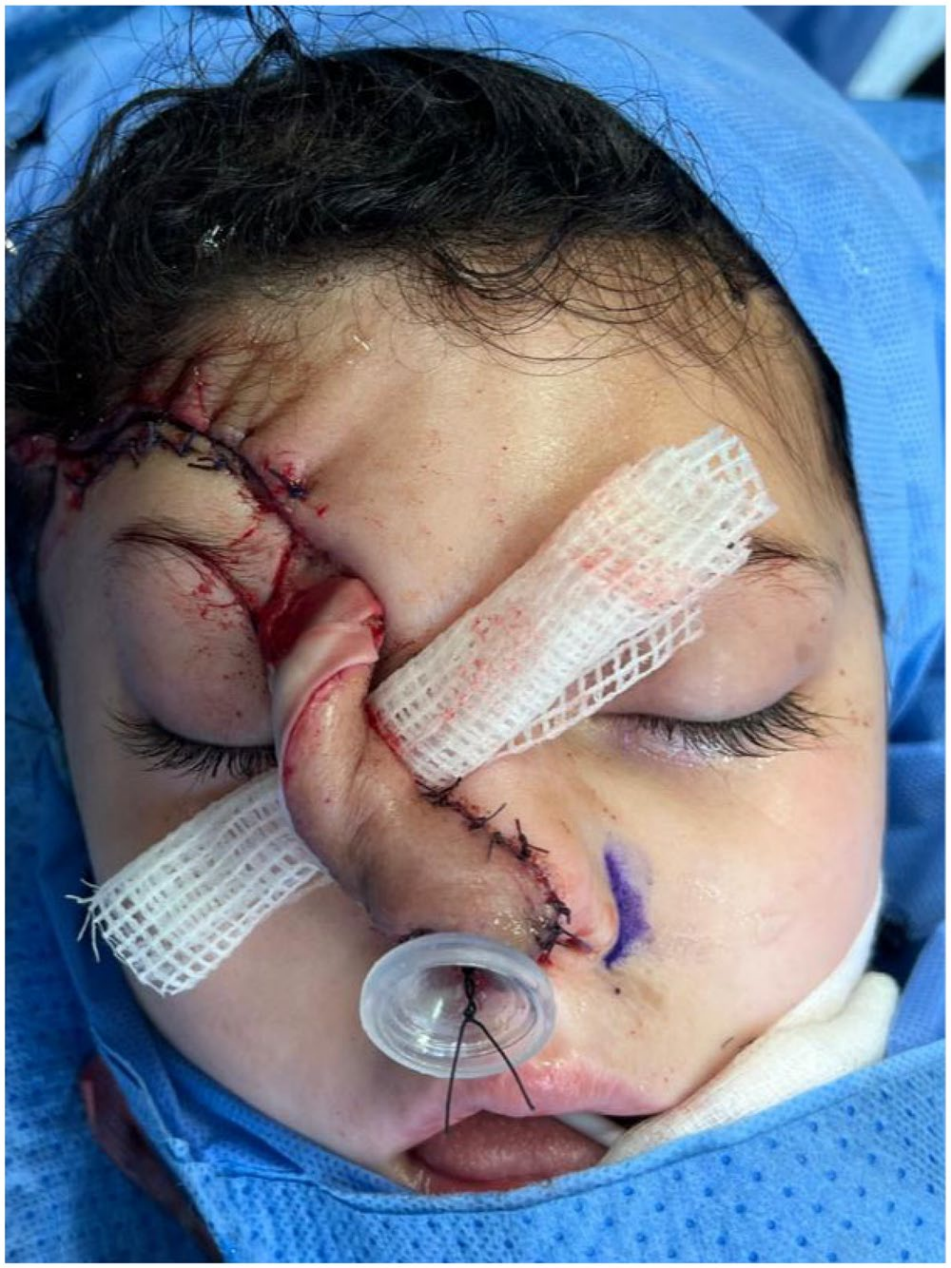
Immediate postoperative appearance following flap transposition and inset for alar reconstruction.

Stage 1 involved elevation of a paramedian forehead flap based on the supratrochlear artery with an initial delay procedure to augment distal perfusion (Figures 2 and 3). Stage 2 consisted of continued flap delay and preparation for transposition (Figure 4). Stage 3 included flap transposition and inset into the surgically modified alar defect. Nasal lining was reconstructed using the remaining native alar skin, which was turned in as a local flap to provide mucosal coverage despite the presence of recipient-site fibrosis. Matriderm, a bilayer collagen-elastin dermal matrix, was applied to the undersurface of the forehead flap to reduce wound burden and control oozing; it was subsequently excised along with excess skin during later stages (Figure 5). Stage 4 comprised reshaping of the flap with the pedicle still attached. A banner-type extension was fashioned from the attached pedicle to adjust alar contour and volume, with contralateral measurements guiding symmetry. Early distal tip congestion was observed and successfully managed with topical nitroglycerin patches. Stage 5 involved complete flap division, final inset, scar revision of the forehead donor site, and use of the proximal pedicle tissue for eyebrow reconstruction.

Alar cartilage grafting was not performed during this reconstructive sequence. Given that the contralateral ala is relatively thin, the decision was made to avoid cartilage grafting to prevent asymmetric bulking of the reconstructed side and achieve a better thickness match. This approach, while departing from classical teaching, was considered appropriate for this patient; cartilage grafting remains an option should functional or structural concerns arise during long-term follow-up.

Intraoperative indocyanine green (ICG) fluorescence angiography and handheld Doppler assessment were used at each stage as confirmatory adjuncts to clinical examination, providing reassurance of adequate flap perfusion in this high-risk patient. ICG findings corroborated clinical assessment but did not alter the operative plan. A summary of the staged reconstructive approach is presented in Table 1.

**Table 1.** Staged surgical reconstruction and outcomes.

Stage	Procedure	Key details	Outcomes
Stage 1	Delayed paramedian forehead flap elevation	Initial expansion to recruit donor skin; flap delay to improve vascularity	Flap raised successfully; good perfusion confirmed with Intraoperative indocyanine green fluorescence angiography /doppler
Stage 2	Continued flap delay and preparation for transposition	Additional delay to augment distal perfusion; preparation of recipient site	Adequate vascular augmentation achieved
Stage 3	Advancement and inset into surgically modified alar defect using remaining alar skin as turn-in flap for lining	Flap advanced; Matriderm applied; VAC dressing placed	Stable flap with good contour; no complications
Stage 4	Reshaping of flap with pedicle still attached; banner flap created to adjust alar contour	Flap reshaped with pedicle still attached; banner flap created for alar contour adjustment; contralateral ala used for symmetry measurements	Reconstructed ala maintained perfusion; improved contour
Stage 5	Final flap division and scar revision of forehead donor site	Flap completely inset; proximal portion used for eyebrow reconstruction	Good perfusion, improved nasal airway, satisfactory cosmetic result

### Postoperative course

The patient tolerated all procedures well under general anesthesia. She remained afebrile, hemodynamically stable, and without major complications. Postoperative monitoring showed excellent flap viability with brisk capillary refill and normal color.

### Outcome and follow-up

**Figure 6.**
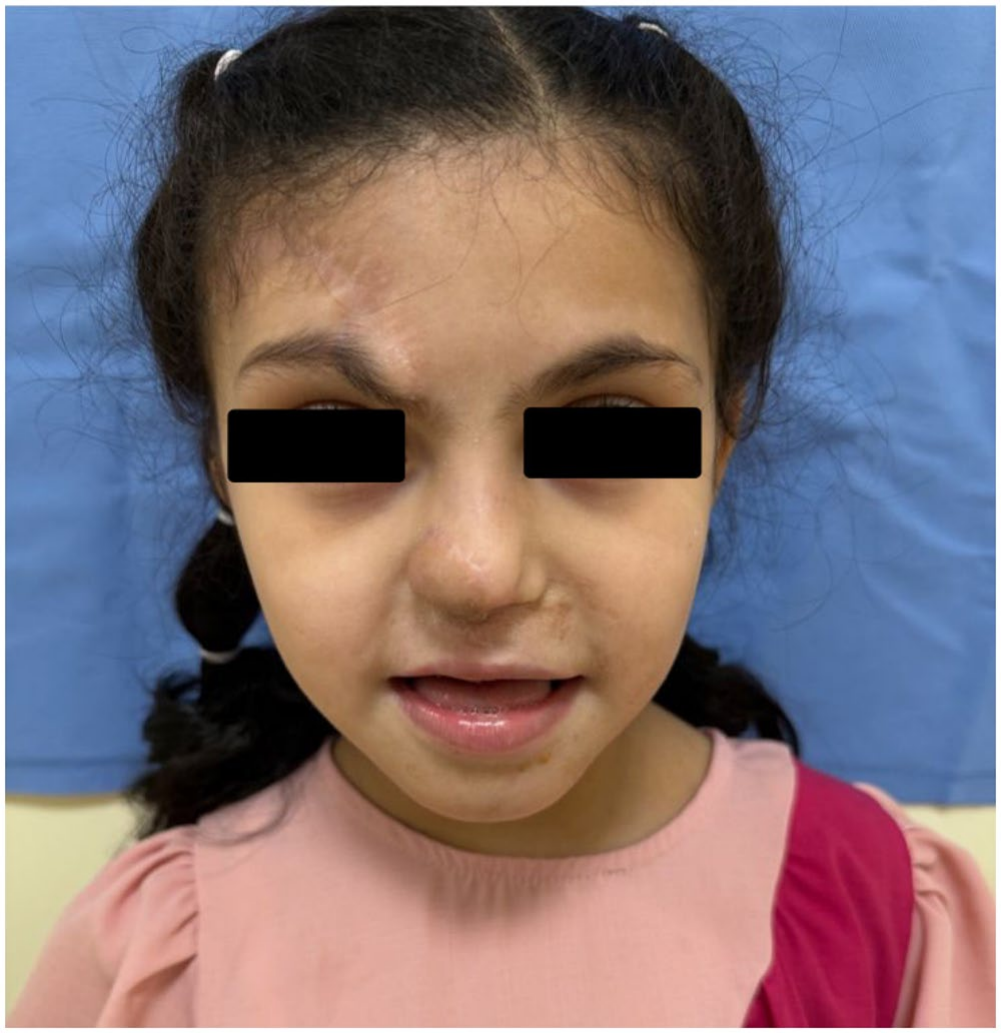
Late postoperative follow-up demonstrating a well-perfused paramedian forehead flap with restoration of the right alar subunit.

At last follow-up, the forehead flap demonstrated healthy perfusion and stable contour. Breathing improved significantly, and parents expressed satisfaction with both functional and cosmetic outcomes (Figure 6). Future stages are planned, including flap defatting, correction of color mismatches with laser hair removal, PICO laser, and tranexamic acid–based depigmenting injections. The patient continued antiviral prophylaxis and multidisciplinary follow-up. This case was managed in accordance with the CARE (CAse REport) guidelines. Informed written consent for participation and publication of clinical details and images was obtained from the patient’s parent/legal guardian prior to inclusion in this report.

## Discussion

Large nasal defects in children are rare, and their reconstruction is particularly challenging. The complexity stems from limited donor tissue, anticipated craniofacial growth, and the psychological and functional impact of facial deformity at a young age [1,2]. While small defects may be addressed with local flaps or skin grafts, extensive full-thickness losses require more robust options. The paramedian forehead flap remains the most reliable reconstructive tool in such scenarios due to its robust supratrochlear pedicle, consistent vascularity, and excellent tissue match [3–5]. Although both unilateral and contralateral forehead flaps are options, we chose the ipsilateral flap because of its favorable arc of rotation and donor site concealment. Contralateral flaps are typically reserved when the ipsilateral tissue is scarred or unavailable. A comparison of forehead flap use between adults and children is summarized in Table 2.

**Table 2.** Comparison of paramedian forehead flap in adults vs children.

Feature	Adults	Children
Indications	Oncologic resection (basal cell carcinoma, squamous cell carcinoma), trauma, infection, congenital deformity	Trauma, congenital anomalies, infection (rare, e.g. HSV, *Pseudomonas*), vascular malformations
Frequency	Common, extensively reported in literature	Rare, only case reports and small series
Donor site	Broad forehead skin, adequate laxity	Smaller forehead, limited skin laxity, tighter tissue planes
Technical considerations	Standard flap design based on supratrochlear artery; well-established algorithms (subunit principle)	Requires tissue expansion or prelamination in many cases; careful staging to avoid distortion of growing structures
Growth concerns	Minimal effect (adult facial skeleton fully developed)	Potential long-term impact on craniofacial growth and scar maturation; need for long-term follow-up
Psychosocial factors	Primarily aesthetic and functional	Both functional (airway, feeding, speech) and high psychosocial impact due to young age
Reported outcomes	High reliability, excellent aesthetic and functional results	Fewer reports, but successful reconstructions described; outcomes generally favorable with staged techniques
Complications	Flap congestion, partial necrosis, contour deformities	Higher risk of donor-site morbidity, limited tissue availability; need for meticulous planning

Forehead flap use in pediatric patients has been reported only infrequently. The smaller forehead size, limited skin laxity, and concerns about potential disturbance of facial growth have limited its adoption [4]. Nonetheless, several reports have demonstrated that forehead flaps in children can yield favorable outcomes without long-term compromise, particularly when carefully staged [2,6]. Our case supports this observation, showing successful staged reconstruction with acceptable donor and recipient site results. Secondary refinements, including flap defatting and contour adjustments, are planned as the soft-tissue reconstruction matures. Cartilage grafting was deliberately avoided given the thin contralateral ala, as adding a graft layer would risk asymmetric bulking; this option remains available should structural support become necessary.

Several reconstructive alternatives to the paramedian forehead flap exist for nasal defects and warrant consideration. Local flaps, including bilobed flaps, nasolabial flaps, and dorsal nasal rotation flaps, are useful for small-to-moderate defects but are limited by their arc of rotation and tissue availability, making them unsuitable for full-thickness, multi-subunit losses [3,4]. Full-thickness skin grafts may reconstruct superficial defects but lack the bulk and vascularity required for composite tissue replacement and often result in poor color match and contraction [5]. Composite auricular grafts can address small alar rim defects but are inadequate for larger reconstructions. Free tissue transfer, such as the radial forearm free flap, offers versatility and can be thinned for nasal reconstruction; however, it requires microvascular expertise, involves significant donor-site morbidity, and is technically demanding in young children with small-caliber vessels [2,6]. Prosthetic reconstruction is a non-surgical alternative but is generally considered a last resort due to issues with retention, maintenance, and psychosocial acceptance, particularly in pediatric patients. In our case, the extensive full-thickness defect involving skin, cartilage, and lining, combined with the patient’s young age and immunocompromised status, made the paramedian forehead flap the most reliable option, offering robust vascularity, adequate tissue volume, and excellent color and texture match.

Infectious etiologies as causes of large pediatric nasal defects are rare. In our patient, recurrent HSV infection in the context of an undiagnosed immunodeficiency played a central role in tissue necrosis. HSV is well known to cause mucocutaneous disease, but destructive necrotizing lesions are rare in immunocompromised children [7,8]. Repeated bacterial superinfections, including *Pseudomonas* and *Staphylococcus* species, likely compounded the tissue loss. This underscores the importance of multidisciplinary management: plastic surgeons must work closely with infectious disease specialists to optimize antiviral prophylaxis, as well as with pediatric neurologists to manage seizure disorder and systemic comorbidities.

Our case also highlights several important technical considerations. The reconstruction was performed in multiple stages, including flap delay, prelamination, and inset. This incremental approach allowed for adequate donor skin recruitment, ensured robust perfusion, and improved the contour of the reconstructed ala. Intraoperative indocyanine green fluorescence angiography and Doppler assessment provided confirmatory reassurance of flap vascularity at each stage. The child tolerated all procedures well, with improved nasal airway patency and a satisfactory aesthetic outcome at follow-up. Table 3 presents a summary of the narrative review of comparable cases documented in the literature. It is important to acknowledge that the cases summarized in our narrative review are etiologically heterogeneous, including trauma (predominantly dog bites), congenital anomalies, and bacterial infections. HSV-related necrotizing nasal defects in immunocompromised children remain exceedingly rare, and to our knowledge, no directly comparable case has been reported in the literature. Therefore, rather than confirming established paradigms, our case expands existing indications for pediatric forehead flap reconstruction into the domain of viral-mediated tissue necrosis in the setting of primary immunodeficiency. This novel context required modifications in staging, perioperative management, and reconstructive sequencing that may not be generalizable to immunocompetent patients with traumatic or congenital defects.

**Table 3.** Selected pediatric nasal reconstructions using forehead flap techniques.

Author, year	Age/sex	Etiology	Defect and key findings	Technique and stages	Outcome/follow-up	Notes
Giugliano et al. 2004 [10]	≤10 y (series, *n* = 10)	Trauma (40%, mainly dog bites), congenital anomalies, and benign skin tumors	Involvement of nasal tip, dorsum, and ala with partial or full-thickness loss; >50% subunit loss enlarged for complete reconstruction	Standard paramedian forehead flap; staged; expansion “in special cases”	Good cosmetic outcomes by objective and subjective grading	Early pediatric series establishing feasibility and subunit principles in children.
Kadlub et al. 2008 [11]	2 years / Male (presented at 15 months)	Subtotal nasal amputation secondary to dog bite	Near-total nasal tissue loss after failed microsurgical replantation	Staged paramedian forehead flap with auricular cartilage graft for structural support; performed at age 2 after initial wound stabilization	Good early functional and aesthetic outcome; satisfactory color and texture match	Among youngest reported; highlights timing considerations in infants.
Exner et al. 2010 [12]	4 months / Female	Necrosis following congenital hemangioma	Subtotal nasal tissue loss involving dorsum, columella, and alar cartilages after vascular tumor necrosis and infection	Immediate reconstruction using right paramedian forehead flap based on supratrochlear artery; pedicle division at 2 months; later refinements: ear composite graft at 19 months, additional ear cartilage graft at 9 years, minor debulking and refinement at 18–20 years	Excellent long-term nasal growth and function with maintained contour and color match; follow-up 20 years	Oldest long-term documented pediatric forehead flap case
Ramanathan et al. 2013 [13]	Pediatric patients (*n* = 9; mean age not specified)	Congenital nasal clefts (Tessier 0–3), including partial arhinia and alar field defects	Multisubunit nasal clefts involving dorsum, alae, and columella; often associated with low hairline and craniofacial anomalies	Three-stage reconstruction using expanded paramedian forehead flap (bilobed/trilobed based on supratrochlear artery); tissue expansion (70–250 mL) over 3 weeks	Good esthetic and functional outcomes; minimal donor-site scarring; tension-free closure	Demonstrates versatility of expanded forehead flap in congenital nasal clefts
Al-Hashmi et al. 2017 [14]	2.5 years / Female	Pseudomonas aeruginosa necrotizing fasciitis	Full-thickness necrosis of left ala, one-third of columella, soft triangle, nostril sill, and upper lip after rapidly progressive infection	Immediate contralateral paramedian forehead flap based on right supratrochlear vessels; conchal cartilage graft for alar support; flap thinned twice, then divided at 3 months post-op	Flap survived completely; patent nostril; good early contour and symmetry; mild edema resolved; family satisfied with outcome	Infectious etiology similar to yours; underscores urgency and reliability.

The limitations of forehead flap reconstruction in children remain important to acknowledge. Long-term surveillance is required to monitor for potential growth-related asymmetry, scar contracture, or donor-site contour deformity [4]. Nevertheless, when faced with extensive defects, particularly in medically complex patients, forehead flap reconstruction remains the gold standard.

The classical paramedian forehead flap reconstruction, as described by Menick, typically follows a two- or three-stage paradigm: initial flap elevation and transposition, followed by intermediate revision if needed, and final pedicle division [4]. In our patient, rather than pre-planning an extended protocol, we adopted an adaptive staging approach, adding stages as clinical circumstances required. This “cut as you go” philosophy allowed flexibility to respond to the patient’s healing, immunologic status, and tissue quality at each interval. The patient’s undiagnosed primary immunodeficiency, young age, and recipient-site fibrosis from recurrent HSV-related necrosis necessitated this individualized approach. Each stage addressed specific needs – vascular augmentation, soft-tissue contouring, or refinement – with interval assessment of wound healing and immunologic stability before proceeding. This patient-specific modification illustrates that the forehead flap paradigm can be adapted to complex pediatric hosts, though it requires close multidisciplinary coordination and acceptance of a potentially prolonged reconstructive timeline.

## Conclusion

This case suggests that staged paramedian forehead flap reconstruction may be safely performed in young children with extensive nasal defects, even in the complex setting of undiagnosed primary immunodeficiency and recurrent HSV-related tissue necrosis. Rather than following a predetermined extended protocol, our adaptive staging approach – adding stages as clinical needs arose – achieved satisfactory functional and aesthetic outcomes at intermediate follow-up. However, as a single-case experience, these findings should be interpreted with caution and cannot be broadly generalized. Key elements contributing to success included flexible staging responsive to patient needs, continuous antiviral prophylaxis, intraoperative perfusion monitoring, and close multidisciplinary coordination. Long-term follow-up is essential to monitor growth-related changes and assess the durability of reconstruction.
